# The imbalance of Th17/Treg cells is involved in the progression of nonalcoholic fatty liver disease in mice

**DOI:** 10.1186/s12865-017-0215-y

**Published:** 2017-06-24

**Authors:** Beihui He, Liyan Wu, Wei Xie, Yitong Shao, Jianping Jiang, Zhenzhong Zhao, Maoxiang Yan, Zhiyun Chen, Dawei Cui

**Affiliations:** 1 Laboratory of Digestive Disease, the First Affiliated Hospital of Zhejiang Chinese Medical University, 54,Youdian Road, Hangzhou, 310006 Zhejiang Province People’s Republic of China; 2 Department of Equipment, the Third People’s Hospital of Hangzhou, 38,Westlake Road, Hangzhou, 310009 People’s Republic of China; 3 The University of Melbourne, Melbourne, VIC 3000 Australia; 4 Department of Preparation Center, the First Affiliated Hospital of Zhejiang Chinese Medical University, 54,Youdian Road, Hangzhou, 310006 Zhejiang Province People’s Republic of China; 5 Department of Gastroenterology, the Second People’s Hospital of Yuhang District of Hangzhou, 80,Anle Road, Yuhang District of Hangzhou, 311100 People’s Republic of China; 6 Department of Laboratory Medicine, First Affiliated Hospital, College of Medicine, Zhejiang University, Hangzhou, 310003 Zhejiang People’s Republic of China

**Keywords:** Nonalcoholic fatty liver disease, Th17 cells, Treg cells

## Abstract

**Background:**

Non-alcoholic fatty liver disease (NAFLD) is a common, chronic liver disease worldwide. Recent studies have shown that T helper (Th) 17 and regulatory T (Treg) cells play critical roles in various disorders of liver inflammation. Here, we explored the value of polyene phosphatidylcholine capsules (PPC) for regulating the imbalance of Th17/Treg cells in the pathogenesis of mice with NAFLD.

**Methods:**

C57BL/6 mice were randomly divided into three groups as follows:normal diet (ND), high-fat diet (HF),and HF plus PPC(HF + PPC). The frequencies of splenic Th17 and Treg cells were measured by flow cytometry, and their related cytokines were analyzed by CBA and real-time PCR.

**Results:**

At the end of 24 weeks, mice in the HF group had a higher frequency of intrahepatic Th17 cells,and a lower proportion of Treg cells compared with the ND group. The levels of Th17 cell-related cytokines (IL-6, IL-17 and IL-23) in serum and in liver tisse were increased,and the hepatic mRNA levels of RORγt, STAT3 and IL-6 were also increased. By contrast,the FoxP3 mRNA level was decreased in the HF group. Moreover, significant pathological and biochemical changes in the liver, as well as serum biochemical changes, were found in mice with NAFLD. Interestingly, following treatment with PPC, the levels of liver inflammation,frequencies of Th17/Treg cells and associated cytokines,and biochemical data were significantly altered.

**Conclusion:**

These findings demonstrate a critical role for PPC in partially attenuating liver inflammatory responses in mice with NAFLD that involves the imbalance of Treg/Th17 cells and associated cytokines.

## Background

Non-alcoholic fatty liver disease (NAFLD),characterized by steatosis, lobular inflammation and hepatocellular ballooning,comprises a spectrum of liver disorders,including simple fatty liver,non-alcoholic steatohepatitis (NASH),hepatic fibrosis,and cirrhosis. NAFLD has become one of the most common chronic diseases worldwide,affecting approximately one-third of population [1–3]. Approximately one-third of patients with NASH have an increased risk of developing liver fibrosis, cirrhosis and hepatocellular carcinoma [3, 4]. The mechanisms mediating these liver disorders,from simple liver steatosis to NASH,remain unclear. Lipopolysaccharide (LPS), oxidative stress, cytokines and other pro-inflammatory mediators may be involved in imposing a ‘second hit’ during the transitional process [4, 5]. Hepatic inflammation is one of the most pronounced features of NASH, and hepatic immune responses play critical roles in the pathogenesis of NASH and other progressive diseases [5, 6]. It is believed that an imbalance between anti-inflammatory T helper type 2 (Th2) cytokines and pro-inflammatory Th1 cytokines is responsible for the development of NASH [6, 7].

Aside from the Th1/Th2 paradigm, recent studies have demonstrated the crucial impact of Th17 cells on hepatic inflammation [8, 9]. Th17 cells express IL-17, IL-17 F, IL-21 and IL-22 cytokines and mediate potent inflammatory immune responses [10]. Accumulating evidence has shown that Th17 cells, and the associated cytokine IL-17, may promote a pro-inflammatory state in chronic viral hepatitis, autoimmune liver diseases, alcoholic liver disease and hepatocellular carcinoma [11–14].By contrast,Treg cells have anti-inflammatory functions and confer protection against liver inflammation [12, 13]. The reciprocal relationship between Treg cells and Th17 cells represents a delicate balance between tolerance and elicitation of immune responses [12]. Previously, we had demonstrated that the proportion of Treg cells was significantly lower in patients with non-alcoholic liver disease [15]. Furthermore, we have focused on the imbalance between Treg and Th17 cells in the development of NAFLD by using a mouse model, which may provide significant understanding of NAFLD-associated inflammatory processes.

In this study, we report that the frequencies of Th17 cells in the high fat diet (HF) group were higher than those in the normal diet (ND) group. Conversely, the frequencies of Treg cells in the HF group were lower than those in the ND group. The levels of Th17 and Treg cell-associated cytokines in the serum and liver were changed in the HF group compared with the ND group. Additionally, hepatic mRNA levels of Th17 cell-related genes, including RORγt, STAT3 and IL-6, were increased,whereas the level of FoxP3 mRNA was decreased in the HF group. Moreover, significant pathological changes in the liver were found,and biochemical changes in the liver and sera were also observed in mice with NAFLD. Interestingly, after treatment with polyene phosphatidylcholine capsules (PPC), the liver inflammation,Th17/Treg cell frequencies,and biochemical data were significantly changed in this study. Taken together,an imbalance between Th17/Treg cells and associated cytokines may be involved in the pathological development of NAFLD, and PPC can attenuate the inflammatory response.

## Methods

### Animal experiments

A total of 80 male C57BL/6 mice (age 6 weeks, each weighing 18–20 g) were purchased from the Shanghai SLAC Laboratory Animal Co.,Ltd.(Shanghai, China) and fed under pathogen-free conditions in the animal facility of Zhejiang Chinese Medical University [certification NO. SCXK (HU) 2007–0005]. Similar to a previous report [3], mice were randomly divided into three treatment groups as follows: an ND group (*n* = 30) (ND, 8% rice bran, 51% maize, 30% soybean powder, 3% bone powder, 1.3% multivitamin, and 6.7% mineral), an HF group (*n* = 30) (HF,75% ND, 2% cholesterol, 15% lard and 8% yolk powder), and an HF + PPC group (*n* = 20). Mice in the HF + PPC group were fed the HF diet with an additional intragastric administration of PPC (PPC, 195.40 mg/kg/mouse, Sanofi, Beijing, China) at the 7th week. The mice were kept at room temperature (21 °C) in a light-controlled environment with ad libitum access to food and water. All animal experiments were approved by the Institutional Animal Care and Use Committee of the First Affiliated Hospital of Zhejiang Chinese Medical University and were conducted in accordance with the National Research Council Guide for Care and Use of Laboratory Animals. The blood was collected by retroorbital route under anesthesia.

### Pathological changes in mice with NAFLD

To determine whether cellular and biochemical changes are consistent with the development of steatohepatitis and whether a possible reversal of the disease process may occur after PPC treatment, we randomly selected ten mice from the ND and HF groups at the 8th, 16th, and 24th weeks and randomly selected ten mice from the HF + PPC group at the 16th and 24th weeks. These mice were sacrificed to obtain liver and serum samples for further study. Liver tissues were stained either with H&E stain (hematoxylin and eosin) or Masson’s trichrome stain and subsequently observed using a light microscope (Olympus, CX41, Japan).

### Treg and Th17 cell frequency analysis by flow cytometry

Splenic lymphocytes from mice were isolated according to a previously reported method [14], and these cells were stained with APC-conjugated anti-mouse CD25 and FITC-conjugated anti-mouse CD4 or isotypes (eBioscience, USA) for 20 min at 4 °C. These cells were washed twice, fixed, permeabilized and then stained with PE-conjugated anti-mouse Foxp3 for analysis of Treg subpopulations. To detect splenic Th17 cells, splenocytes were stimulated with 50 ng/ml phorbol 12-myristate 13-acetate (PMA) (BioVision, Mountain View, CA, USA), 1 μg/ml ionomycin (Enzo Life Sciences, Farmingdale, NY, USA) and 500 ng/ml monensin (eBscience, San Diego, CA, USA) for 4 h and were stained with FITC-conjugated anti-mouse CD4 and PE-conjugated anti-mouse IL-17 antibodies. Acquisitions were performed using a FACS Canto II flow cytometer (BD, USA). Data were analyzed based on the percentages of Th17 cells and Treg cells.

### Serum biochemistry and cytokines of mice with NAFLD

Serum from mice was collected at 8, 16 and 24 weeks. Alanine aminotransferase (ALT), aspartate aminotransferase (AST), triglyceride (TG) and total cholesterol (CHOL) levels were measured using a multi-channel auto-analyzer (Hitachi 7180, Japan). Levels of serum cytokines, including TNF-α, IL-6, IL-17, IL-23, and TGF-β, were also measured by ELISA (eBioscience, San Diego, USA).

### RNA extraction and real-time PCR assay

RNA was extracted from the liver of mice using Trizol reagent, and complementary DNA synthesis was performed according to the manufacturer’s instructions. PCR assays were conducted using an ABI7900 Real Time PCR machine and SYBR Premix Ex TaqTM (Takara, Shiga, Japan). The primer sequences of IL-6, retinoid-related orphan receptor RORγt, STAT3, FoxP3 and β-actin genes are presented in Table 1.

**Table 1:** Primer sequences of T helper type 17 (Th17)-related mouse cytokines

Name		Primers (5’ ➝ 3’)	Product (bp)
β-actin	Sense	AGAGGGAAATCGTGCGTGAC	138 bp
	Antisense	CAATAGTGATGACCTGGCCGT	
IL-6	Sense	ACAACCACGGCCTTCCCTACTT	139 bp
	Antisense	CACGATTTCCCAGAGAACATGTG	
RORγt	Sense	GCCGCGGAGCAGACACACTT	176 bp
	Antisense	GGAGGCCCCCTGGACCTCTG	
Foxp3	Sense	CAGGAGAAAGCGGATACCAAATG	176 bp
	Antisense	ATCTGTGAGGACTACCGAGCC	
STAT3	Sense	ACCTCCAGGACGACTTTGAT	203 bp
	Antisense	TGTCTTCTGCACGTACTCCA	

The housekeeping gene β-action was used as an internal control, and gene-specific mRNA expression was normalized against β-action expression. Relative quantification by the 2^-△△Ct^ method was realized by comparing to control groups

### Statistical analysis

Data are expressed as the means and standard deviations (mean ± SD). Data were analyzed by ANOVA. All statistical analyses were performed using the statistical SPSS software (version 17.0, SPSS Inc., USA). *P*-values <0.05 were considered statistically significant.

## Results

### Pathologic changes of liver tissue in NAFLD mice

**Fig. 1:**
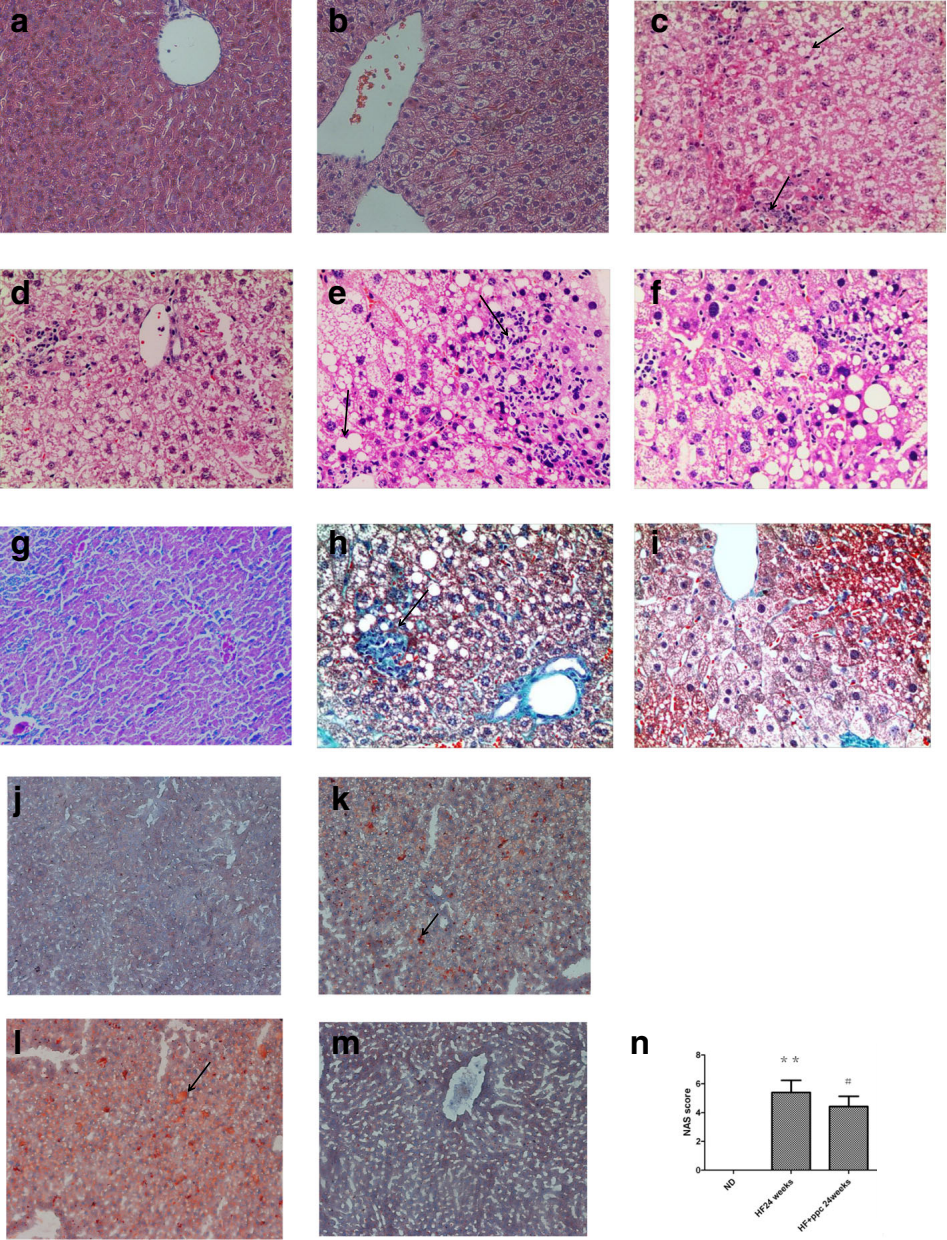
Pathologic changes of liver tissue in mice. **a** ND group at 8 weeks; **b** HF group at 8 weeks; **c** HF group at 16 weeks; **d** HF + PPC group at 16 weeks; **e** HF group at 24 weeks; **f** HF + PPC group at 24 weeks; **g** ND group at 24 weeks; **h** HF group at 24 weeks; **i** HF + PPC group at 24 weeks; **j** ND group at 8 weeks; **k** HF group at 8 weeks; **l** HF group at 16 weeks; **m** HF + PPC group at 16 weeks; **n** NAS score (**a**-**f**, H&E stain, magnification × 200; **g**-**i** Masson’s trichrome staining, magnification × 200; **j**-**m** Oil Red O staining,magnification × 100). **n** HF vs. ND: **, *P* < 0.01. HF + PPC vs. HF: #, *P* < 0.05

Compared with the ND group, moderate liver cell steatosis and mild lobular inflammatory cell infiltration were observed in mice from the HF group at 8 weeks (Fig. 1a and b). At 16 weeks, severe fatty degeneration, lobular infiltration of inflammatory cells and significantly increased focal necrosis were found in mice from the HF group compared with that at 8 weeks (Fig. 1c and d). The mice in the HF group developed severe steatosis, further increased lobular inflammation and necrosis, and perisinusoidal fibrosis of varying degrees at 24 weeks (Fig. 1e and g). After treatment with PPC, murine liver cells displayed a lower degree of inflammation despite the mice still being fed a high-fat diet (Fig. 1f and i). The average NAS scores were significantly reduced in HF + PPC group at 24 weeks (*P* < 0.05) (Fig. 1n). Oil red O staining provided a much more indicative view of the fat accumulation in HF groups at 8 and 16 weeks,and the fat accumulation could be significantly ameliorated by PPC treatment (Fig. 1j, k, l and m). Therefore, microscopic pathological changes in line with NASH were observed in mice fed a high-fat diet, and these pathological changes inflammatory changes could be significantly ameliorated by PPC treatment.

### Biochemical changes in serum and liver in NAFLD mice

**Fig. 2:**
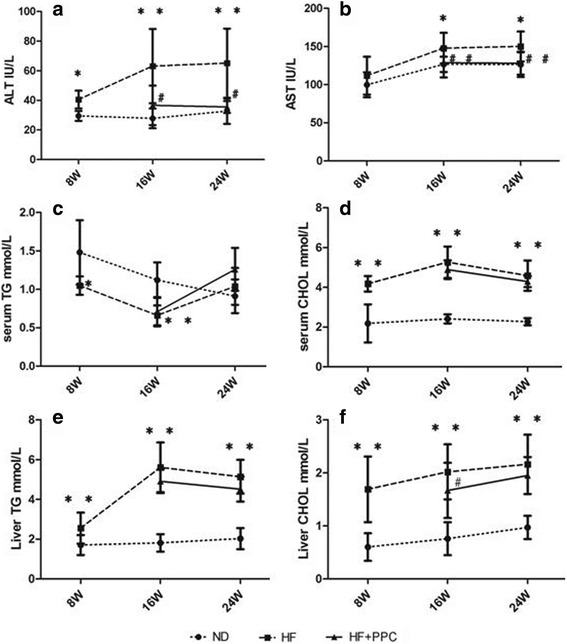
Dynamic changes of ALT, AST, TG, and CHOL in serum and liver TG and CHOL in mice with NAFLD. **a** serum ALT levels; **b** serum AST levels; **c** serum TG levels; **d** serum CHOL levels; **e** liver TG levels; **f** liver CHOL levels. HF vs. ND: *, *P* < 0.05; **, *P* < 0.01. HF + PPC vs. HF: #, *P* < 0.05; ##, *P* < 0.01

Serum AST levels from mice in the HF group were higher at 8 weeks than those in the ND group (*P* < 0.05) and were dramatically increased at 16 and 24 weeks compared with the ND group (*P* < 0.01). An increasing trend in the serum ALT level was observed in the HF group at 8 weeks, although there was no statistically significant difference between the HF and ND groups (*P* > 0.05). Serum ALT levels in the HF group were increased at both 16 and 24 weeks compared with those in the ND group (*P* < 0.05). After the intervention with PPC, at 16 and 24 weeks, both the AST and ALT levels were remarkably decreased compared with the HF group (*P* < 0.05 and *P* < 0.01, respectively) (Fig. 2a and b). The serum CHOL and liver TG levels in the HF group at 8, 16 and 24 weeks were significantly higher than those in the ND group (*P* < 0.01), with a peak at 16 weeks (Fig. 2d and e). The serum TG levels in the HF group at 8 and 16 weeks were decreased in contrast to those in the ND group (*P* < 0.05 and *P* < 0.01, respectively) (Fig. 2c). After treatment with PPC, the liver CHOL levels in mice at 16 weeks notably declined (*P* < 0.05), whereas no significant difference was observed at 8 and 24 weeks compared with the HF group (*P* > 0.05) (Fig. 2f).

### Frequencies of Treg cells and Th17 cells in NAFLD mice

**Fig. 3:**
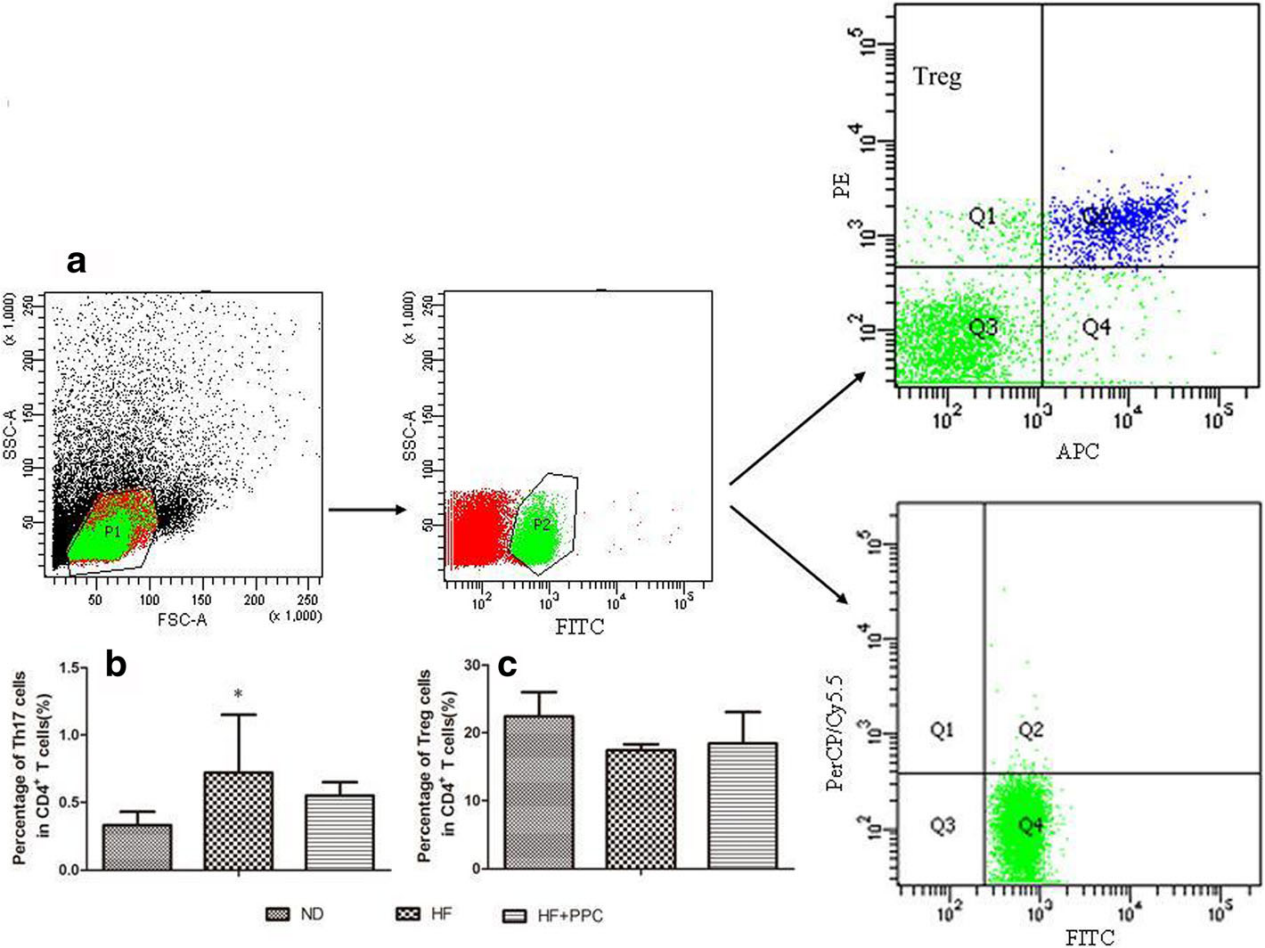
Frequency changes of splenic Treg and Th17 cells in mice with NAFLD. **a** frequencies of Treg and Th17 cells were detected by flow cytometry; **b** frequency of Th17 cells; **c** frequency of Treg cells in the three groups. HF vs. ND: *, *P* < 0.05; **, *P* < 0.01. HF + PPC vs. HF: #, *P* < 0.05; ##, *P* < 0.01

The frequencies of splenic Treg cells and Th17 cells in mice with NAFLD were analyzed by flow cytometry (Fig. 3a). The frequencies of splenic Th17 cells in the HF group were significantly increased compared with the ND group at 24 weeks (*P* < 0.05) (Fig. 3b). Treg cells had a tendency to decrease at 24 weeks, but no statistical significance was observed (*P* > 0.05) (Fig. 3b). After the intervention with PPC, we observed a decrease in the Th17 cell population and an increase in the Treg cell population in the HF + PPC group compared with the HF group at 24 weeks, but these trends were not significant (*P* > 0.05) (Fig. 3a and b).

### Serum cytokines in NAFLD mice

**Fig. 4:**
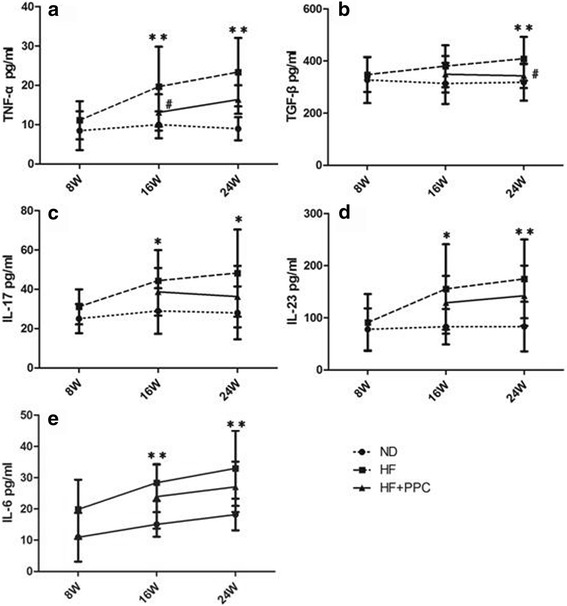
Dynamic changes of Th17 and Treg cell-related cytokines in serum in mice with NAFLD. **a** serum TNF-α levels; **b** serum TGF-β levels; **c** serum IL-17 levels; **d** serum IL-23 levels; **e** serum IL-6 levels. HF vs. ND: *, *P* < 0.05; **, *P* < 0.01. HF + PPC vs. HF: #, *P* < 0.05; ##, *P* < 0.01

Cytokine (TNF-α, IL-6, IL-17, and IL-23) levels gradually increased from 8 to 24 weeks in the HF group (Fig. 4). The levels of the aforementioned cytokines in the HF group were not significantly different compared with the ND group at 8 weeks (*P* > 0.05), whereas the levels at 16 and 24 weeks were significantly increased compared with the ND group (*P* < 0.05 and *P* < 0.01, respectively) (Fig. 4a and c-e). The TGF-β levels in the HF group were only significantly higher at 24 weeks compared with the ND group (*P* < 0.01) (Fig. 4b). After the intervention with PPC, the TNF-α levels in the HF + PPC group at 16 and 24 weeks were significantly decreased compared with the HF group (*P* < 0.05) (Fig. 4a). Meanwhile, the TGF-β levels in the HF + PPC group at 24 weeks were significantly decreased compared with the HF group (*P* < 0.05) (Fig. 4b); however, other cytokines showed no significant difference (*P* > 0.05) (Fig. 4c-e).

### Treg/Th17-related mRNA expression in the liver tissues of NAFLD mice

**Fig. 5:**
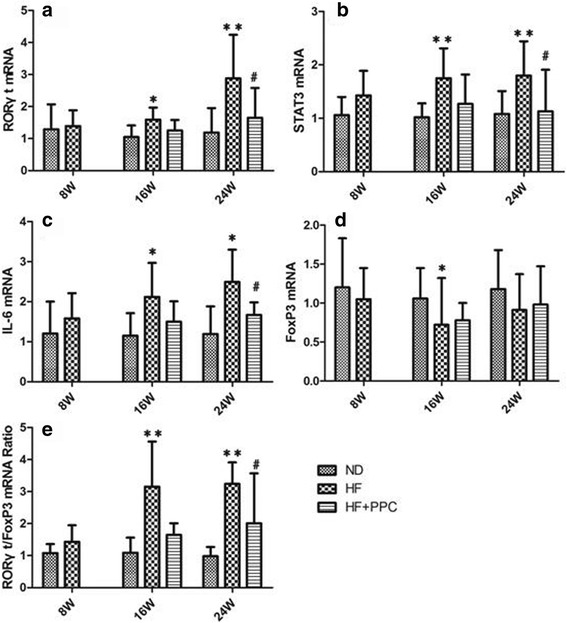
Increased mRNA expression of IL-6, RORγt, STAT3, and FoxP3 in mice with NAFLD. **a** levels of the relative expression of RORγt mRNA in liver; **b** levels of the relative expression of STAT3 mRNA in liver; **c** levels of the relative expression of IL-6 mRNA in liver; **d** levels of the relative expression of FoxP3 mRNA in liver; **e** ratio of RORγt/FoxP3 mRNA in liver from mice with NAFLD. HF vs. ND: *, *P* < 0.05; **, *P* < 0.01. HF + PPC vs. HF: #, *P* < 0.05; ##, *P* < 0.01

The RORγt, STAT3, and IL-6 mRNA levels and RORγt/FoxP3 ratio in the HF group were not significantly different compared with the ND group at 8 weeks (*P* > 0.05); however, they significantly increased compared with the ND group at 16 and 24 weeks (*P* < 0.05 and *P* < 0.01, respectively). The expression levels of RORγt, STAT3, and IL-6 mRNA and RORγt/FoxP3 ratio in the HF + PPC group at 24 weeks were significantly decreased compared with the HF group (*P* < 0.05) (Fig. 5a, b, c and e). The expression of FoxP3 mRNA in the liver tissues in the HF group at 16 weeks was significantly decreased (*P* < 0.05), but no significant difference at 8 or 24 weeks was observed compared with the ND group (*P* > 0.05) (Fig. 5d).

## Discussion

Accumulating evidence indicates that approximately 30% of NAFLD patients may progress to NASH, which is characterized by lobular inflammation and ballooning degeneration, and these patients may further develop liver fibrosis, liver cirrhosis and hepatocellular carcinoma [16]. Understanding the mechanisms by which simple fatty liver develops into NASH is critical. Several risk factors (such as LPS, oxidative stress and inflammatory cytokines) are likely to provide a "second hit", which may contribute to the transformation process from simple steatosis to NASH [4, 5, 17]. Several studies have demonstrated that immune disorders, such as an imbalance of anti-inflammatory cytokines and pro-inflammatory cytokines, are involved in liver inflammation [18, 19]. A previous study suggested that NASH is mainly associated with an imbalance of Th1/Th2 immune responses, with Th1 predominance [20].

Recent reports suggest that Th17 cells, which produce IL-17A, IL-17 F, IL-21 and IL-22, not only mediate the clearance of pathogens but also promote the progression of tissue inflammation and autoimmune diseases [21, 22]. IL-6 and TGF-β cytokines are required for differentiation and proliferation of Th17 cells expressing the transcription factor RORγt. IL-21 and IL-23 cytokines contribute to the differentiation of Th17 cells, and IL-23 promotes maturation in the later stages of differentiation of Th17 cells [20–23]. IL-17, IL-6 and IL-21 cytokines can aggravate the development of autoimmune disease by triggering inflammatory reactions [20, 24]. IL-6 was demonstrated to paralyze the Treg cells’ suppressive effects on Th17 cells and contributed to liver injury by promoting expansion of Th17 cells [24]. STAT3 and RORγt are specific transcription factors responsible for the differentiation of Th17 cells, and the expression of IL-17 mRNA is closely associated with RORγt expression [22]. Treg cells can inhibit inflammation and maintain self-tolerance, and an altered balance of TGF-β and IL-6 may control the switching of Treg or Th17 cells via antagonistic competition of Foxp3 and RORγt [25–27].

The functions of Th17 cells and IL-17 in liver diseases have been extensively explored [13, 26, 28]. Th17 cells are associated with hepatocellular steatosis and inflammatory processes [13]. IL-17 and fatty acids can synergistically stimulate hepatic cells to secrete IL-6, and IL-6 aggravates insulin resistance. As IL-17 increases hepatic inflammation, their cooperation can exacerbate the development of NAFLD [8, 29]. By contrast, CD4^+^CD25^+^Foxp3^+^Treg cells contribute to attenuation of liver inflammatory diseases [30, 31]. Previous studies have shown that the number of hepatic Treg cells was significantly decreased in mice with NAFLD [8], and the percentage of Treg cells and expression of Foxp3 mRNA were also decreased in mice with NASH [32]. Our previous report indicated that the proportion and number of Treg cells were significantly lower in NAFLD patients [15]. Importantly, the Treg/Th17 balance was relevant for NAFLD, and it may predict long-term outcomes in NAFLD patients. In this study, we found that NAFLD mice experienced a transformation process from simple fatty liver disease to fatty hepatitis to the early stages of liver fibrosis. The frequencies of Th17 cells were elevated, with increased mRNA levels of IL-6, RORγt, and STAT3 genes in liver tissues and high concentrations of IL-17 in serum. These findings are in accordance with previous reports [8, 29–32]. PPC, trade name Essentiale®, can partially alleviate liver injury, improve liver enzyme activity, promote the regeneration of liver tissue, and is commonly used in the clinical treatment of NAFLD [15]. In this study, liver inflammation and fibrosis of varying degrees were reduced after treatment with PPC at 16 and 24 weeks. The mRNA expression levels of RORγt, STAT3, and IL-6 genes and the RORγt/FoxP3 ratio were significantly reduced at 24 weeks compared with the HF group. The imbalance of Treg/Th17 also had a distinct correction. According to these findings, we hypothesize that under normal conditions, the ratio of Treg/Th17 cells in the host is balanced. A long-term high-fat diet causes abnormal secretions of murine liver tissue cytokines TGF-β and IL-6 that co-stimulates the further activation of STAT3 and RORγt expression. This process promotes the differentiation of CD4 + T cells to Th17 cells and the release of IL-17, and Th17 cells inhibit the differentiation of Treg cells. The more dominant Th17 cells are involved in liver damage through multiple pathways, exacerbating the development of NAFLD. PPC contribute to the attenuation of liver inflammatory responses in mice with NAFLD that may be involved in changes of the Treg/Th17 cell imbalance and associated cytokines.

## Conclusions

Our results indicate that the imbalance of Treg/Th17 cells plays an important role in the pathological mechanism of NAFLD and that changes of related cytokines might be helpful for the differentiation of Treg/Th17 cells and inflammatory responses. PPC intervention may partially attenuate the inflammatory response by adjusting the imbalance of Th17/Treg cells, thus ameliorating the progression of NAFLD.

## Abbreviations

ALT: Alanine aminotransferase
AST: Aspartate aminotransferase
CHOL: Cholesterol
HF: High-fat diet
HF + PPC: High-fat diet plus polyene phosphatidylcholine capsules
LPS: Lipopolysaccharide
NAFLD: Non-alcoholic fatty liver disease
NASH: Non-alcoholic steatohepatitis
ND: Normal diet
TG: Triglyceride
